# Assessing the Exoproteome of Marine Bacteria, Lesson from a RTX-Toxin Abundantly Secreted by *Phaeobacter Strain DSM 17395*

**DOI:** 10.1371/journal.pone.0089691

**Published:** 2014-02-24

**Authors:** Emie Durighello, Joseph Alexander Christie-Oleza, Jean Armengaud

**Affiliations:** CEA, DSV, IBEB, Lab Biochim System Perturb, Bagnols-sur-Cèze, France; CEA (Atomic and Alternative Energies Commission), France

## Abstract

Bacteria from the *Roseobacter* clade are abundant in surface marine ecosystems as over 10% of bacterial cells in the open ocean and 20% in coastal waters belong to this group. In order to document how these marine bacteria interact with their environment, we analyzed the exoproteome of *Phaeobacter strain DSM 17395.* We grew the strain in marine medium, collected the exoproteome and catalogued its content with high-throughput nanoLC-MS/MS shotgun proteomics. The major component represented 60% of the total protein content but was refractory to either classical proteomic identification or proteogenomics. We *de novo* sequenced this abundant protein with high-resolution tandem mass spectra which turned out being the 53 kDa RTX-toxin ZP_02147451. It comprised a peptidase M10 serralysin domain. We explained its recalcitrance to trypsin proteolysis and proteomic identification by its unusual low number of basic residues. We found this is a conserved trait in RTX-toxins from *Roseobacter* strains which probably explains their persistence in the harsh conditions around bacteria. Comprehensive analysis of exoproteomes from environmental bacteria should take into account this proteolytic recalcitrance.

## Introduction

The extremely high diversity of marine microorganisms was shown ever since the first large metagenomic shotgun analysis carried out on total DNA extracted from filtered seawater samples collected from the Sargasso Sea near Bermuda [1]. Heterotrophic bacterioplankton communities in near-surface marine pelagic environments mostly comprise *Alphaproteobacteria* and *Gammaproteobacteria*, as well as *Sphingobacteria* and *Flavobacteria* (both belonging to the *Bacteroidetes* superphylum). As shown recently with environmental metagenomic data, prominent components belong to the SAR11, *Roseobacter* and SAR86 clades together with the OM60/NOR5 cluster [2]. The *Roseobacter* lineage is a phylogenetically coherent group of *Alphaproteobacteria* comprising up to 20% of marine microbial communities, especially in coastal and polar waters [3]. However, the *Roseobacter* clade is a physiologically heterogeneous group showing a high variety of lithoheterotrophic strategies. Despite a free-living lifestyle, *Roseobacter* strains are also found as planktonic or larger pluricellular symbionts [4].

Today, in-depth analysis of a microbial proteome relies on the use of high-resolution hybrid tandem mass spectrometers coupled to high-pressure liquid chromatography systems able to resolve complex peptide mixtures [5], [6]. A comprehensive list of proteins and their quantitation in several physiological conditions by such shotgun approaches are a rather straightforward method [7], [8]. Exoproteomes comprise the fraction of proteins secreted by cellular systems and proteins arising from cell lysis [9]. These are generally subjected to intense proteolytic activities and most components have a short half-life. Nevertheless, key components involved in nutrient imports, cell motility, signaling and interactions with other organisms should be refractory to proteolysis in order to be efficient. Analysis of bacterial secretomes is an important issue for the discovery of novel bioactive compounds and to determine how organisms interact within their environment. We have shown that microbial exoproteomes can be comprehensively analyzed by high throughput shotgun proteomics [10], [11]. The use of the most recent generation of hybrid tandem mass spectrometers revealed a high diversity of protein virulence factors in the pathogenic *Bacillus cereus* bacteria [11]. To date, only a few exoproteome analyses have been reported on environmental avirulent isolates [10], [12]. In this case, the data obtained helped elucidating the ecological distinctness among strains of the *Roseobacter* clade. Microbial exoproteomes are also insightful for understanding the molecular mechanisms of bacterial colonization of biotic or abiotic surfaces, i.e. bacterial adhesion and biofilm formation [13]. Their exploration gains ground with the current development of proteomic tools for environmental microbiology [14].

*Phaeobacter gallaeciensis* BS107 (previously *Roseobacter gallaeciensis* BS107) is the type species of the *Phaeobacter* genus [15]. This strain was obtained from larval cultures and collectors of the scallop *Pecten maximus* [16]. Other species of the genus have since been described: *Phaeobacter inhibens*, isolated from the German Wadden Sea [15], *Phaeobacter daeponensis*, isolated from a tidal flat of the Yellow Sea at Daepo Beach in Korea [17], and *Phaeobacter arcticus*, a psychrophilic species isolated from marine sediments of the Arctic Ocean [18]. *Phaeobacter inhibens* was in-depth characterized with proteomics and metabolomics approaches highlighting its versatile metabolism [19], [20], [21], [22]. The type species *Phaeobacter strain DSM 17395* was shown to be able to produce a new tropolone derivative, tropodithietic acid, which exhibits strong antibiotic properties against marine bacteria of various taxa and marine algae [23], [24]. Additional studies led to the identification of nine new troponoid compounds belonging to the bacterial roseobacticide family [25]. More recently, it has been shown that *Phaeobacter strain DSM 17395* switches its secreted molecule metabolism to the production of potent and selective algaecides in response to p-coumaric acid, an algal lignin breakdown product that is symptomatic of aging algae. This switch converts *Phaeobacter strain DSM 17395* into an opportunistic pathogen of its algal host [26] and highlights the interest of the study of its secretome. The type strain *Phaeobacter gallaeciensis BS107T* has been deposited at public culture collections worldwide. Recently, it was shown that the Phaeobacter strain DSM 17395, previously annotated as *Phaeobacter gallaeciensis BS107T,* exhibits a much closer affiliation to *Phaeobacter inhibens* DSM 16374T and should thus be allocated to this species [27].

With the intention to further characterize the ecological distinctness of *Roseobacters* members and how they interact with their environment, we analyzed the secreted proteins of *Phaeobacter strain DSM 17395*. For this, we grew the strain in marine medium, collected the exoproteome and catalogued its protein content with high throughput nanoLC-MS/MS shotgun proteomics. Interestingly, the major protein in this exoproteome was recalcitrant to classical proteomic identification. Indeed it appears very abundant on SDS-PAGE migration but no protein was matching this band after proteomic analysis. A *de novo* sequencing strategy identified peptides of a RTX-like toxin which led to re-analyze proteomics data with no-enzyme specificity. This RTX-like toxin was then clearly identified. We found that this protein and its homologues exhibit unusually low content in basic residues. From these results, we proposed a general strategy to obtain more comprehensive exoproteome analyses of environmental bacteria.

## Materials and Methods

### *P. gallaenciensis* Growth and Exoproteome Preparation

Four flasks containing 40 mL of marine broth (Difco) were inoculated with *Phaeobacter strain DSM 17395* cells previously grown on marine agar plates. The cultures were incubated at 30°C under 180 rpm agitation until mid-exponential phase growth (OD_600_ = 0.6). Cultures were centrifuged at 3,000 *g* for 10 min at 20°C and supernatants were carefully filtered through two low protein-binding filters of 0.45 µm (Millex-Hv) and then 0.22 µm (Millex-GV) diameter pore (Millipore) in order to eliminate bacterial cells. Supernatants were frozen prior protein precipitation. Proteins from the supernatant were precipitated with tricholoroacetic acid as previously described [10]. The resulting protein pellets were dissolved into 90 µl of lithium dodecyl sulfate-β-mercaptoethanol protein gel sample buffer (Invitrogen), incubated at 99°C for 5 min and briefly centrifuged prior SDS-PAGE.

### SDS-PAGE and Trypsin-*in gel* Proteolysis

A volume of 20 µl of the concentrated exoproteome of *Phaeobacter strain DSM 17395* was deposited per well onto 10% Bis-Tris NuPAGE gels (Invitrogen). SDS-PAGE was carried out using 1X 3-(N-morpholino) propanesulfonic acid solution (Invitrogen) in a XCell SureLock Mini-cell (Invitrogen) under a constant voltage of 200 V. At first, a long migration was carried out in order to fully resolve the exoproteome according to its molecular weight. For the proteomic shotgun analysis, the entire exoproteome was allowed to enter the polyacrylamide gel in a short migration (3 mm). Both gels were stained with SimplyBlue SafeStain, a ready-to-use Coomassie G-250 stain (Invitrogen). SeeBlue Plus2 (Invitrogen) was used as a molecular weight marker. Densitometry analysis of the long-migrated SDS-PAGE gel was carried out with the molecular imager GS-800 calibrated densitometer (BioRad) and the Quantity One software (version 4.6.9, BioRad) in order to establish the relative abundance of the main protein band. Densitometric relative ratio was obtained by comparing the density signal of each band to the total signal of the whole gel lane. These measurements were done in triplicate. Polyacrylamide gel bands (equivalent in volume to 50 µl) were cut and processed for *in*-gel proteolysis with Trypsin Sequencing Grade (Roche) followed by the ProteaseMax protocol (Promega) as previously described [11].

### NanoLC-MS/MS Analysis

Peptide digests were resolved on an UltiMate 3000 LC system (Dionex-LC Packings) as previously described [28] prior to MS/MS measurements done with a LTQ-Orbitrap XL (ThermoFisher). For the FT/IT procedure, parameters used for the tandem mass spectrometry measurements were those previously described [29]. During the FT/FT procedure, the LC gradient used was a 90 min gradient with aqueous solvent A (0.1% HCOOH) and solvent B (0.1% HCOOH/80% CH_3_CN) developed as follows: 5–60% B in 90 min, 60–90% B in 1 min, 90% B during 10 min, 90–5% B in 1 min and 5% B during 19 min. The “full scan” mass spectrum was measured from *m/z* 300 to 1800 th and the charge states considered were +2 and +3. A scan cycle was started with a high resolution full scan (resolution = 30,000) with the Orbitrap analyzer, followed by a high resolution MS/MS scan of the secondary ions obtained after fragmentation, again with the Orbitrap analyzer but with a slightly lower resolution (resolution = 15,000). A 30 sec dynamic exclusion was set. The activation type used was CID with a standard normalized collision energy set at 35.

### MS/MS Database MASCOT Search and Protein Quantification by Spectral Count

Peak lists were generated with the MASCOT DAEMON software (v2.3.2, Matrix Science) from the LC-MS/MS raw data using default parameters. The MASCOT 2.3.02 search engine (Matrix Science) was used to search all MS/MS spectra against a database corresponding to a complete list of annotated Coding Domain Sequences (CDS) from *Phaeobacter strain DSM 17395* (NCBI RefSeq: NC_018290.1, NC_018291.1, NC_018288.1, and NC_018287.1). Bovine trypsin and the 22 most common keratin contaminants were included as a follow-up. Searches for peptides were first performed with the following parameters: mass tolerance of 5 ppm on the parent ion and 0.5 Da on the MS/MS (FT/IT procedure), static modifications of carboxamidomethylated Cys (+57.0215), and dynamic modification of oxidized Met (+15.9949). High-resolution MS/MS (FT/FT procedure) searches were performed with mass tolerance of 0.1 Da on the MS/MS. To process the data resulting from trypsin proteolysis, the maximum number of missed cleavage was set at 2. All peptide matches with a score above its peptidic identity threshold (set at p<0.01) with the ORF database and rank 1 were filtered with the IRMa 1.28.0 software [30]. A protein was only validated when at least two peptides had been assigned. In a second stage, we performed additional searches for both “semi-trypsin” and “no-enzyme” protease specificities. Protein abundance was evaluated in the shotgun analysis by MS/MS spectral counts as established by Liu and co-workers [31] and previously described [32]. Normalized Spectral count Abundance Factors (NSAF) was calculated as defined by Paoletti et al. [33].

### *De novo* Assignment of MS/MS Spectra

Peak lists were submitted to Peaks Studio 5.3 software (Bioinformatics Solutions Inc). At first, a data refinement step was performed with a quality threshold set at 0.65. *De novo* sequencing was then carried out with the following parameters: a parent mass error tolerance of 5.0 ppm, a fragment mass error tolerance of 0.1 Da, cysteine carbamidomethylation (+57.02) as fixed modification, and methionine oxidation (+15.99) as variable modification. More stringent criteria were applied with a parent mass error tolerance of 2 ppm with high resolution mode for both MS and MS/MS. Successively, trypsin, semi-trypsin and no-enzyme were chosen as enzyme specificities. The list of assigned spectra obtained with MASCOT using *Phaeobacter strain DSM 17395* CDS database was compared with Peaks Studio results for each samples. Precursor mass spectra annotated with the Peaks Studio software but not interpreted with the Mascot engine gave candidates for manual annotation. Results were then sorted by their Average of Local Confidence (ALC) in order to choose the best spectra to annotate.

## Results and Discussion

### The most Abundant Protein within the Exoproteome of *Phaeobacter strain DSM 17395* is Recalcitrant to Standard Proteomic Identification

**Figure 1:**
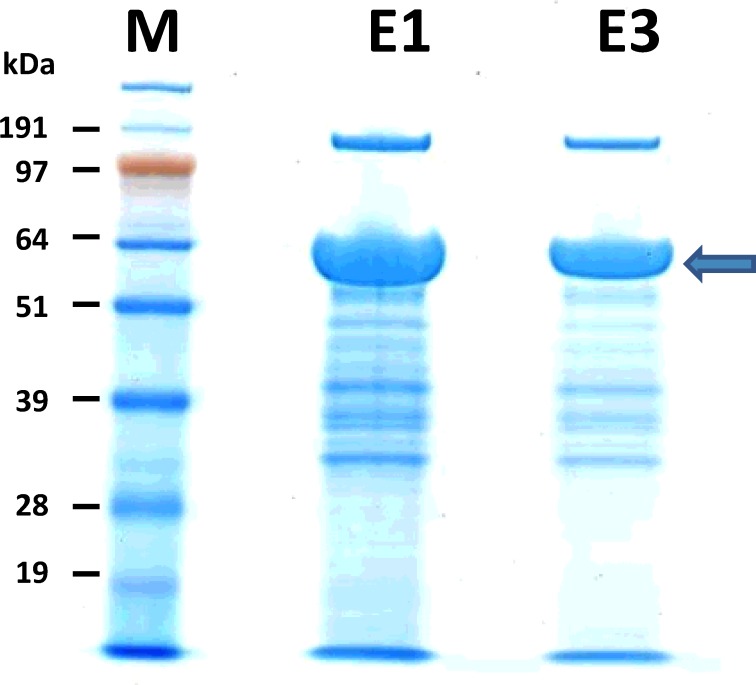
SDS-PAGE of the exoproteome of *Phaeobacter strain DSM 17395*. Exoproteins were resolved by a long migration on a 10% SDS-PAGE and stained with SimplyBlue SafeStain (Invitrogen). Lane **M**: SeeBlue Plus2 molecular weight range marker (Invitrogen). Lane **E1**: *Phaeobacter strain DSM 17395* exoproteome grown in Marine Broth (20 µg). Lane **E2**: *Phaeobacter strain DSM 17395* exoproteome grown in Marine Broth (8 µg). The 55 kDa major component is indicated with an arrow.

The resulting exoproteome of the marine bacterium *Phaeobacter strain DSM 17395* when grown in Marine Broth was collected and resolved by SDS-PAGE. Remarkably, an abundant protein band migrating at a molecular weight of about 55 kDa was observed in the protein profile. This 55 kDa band accounts for 60 (±2) % of the total exoproteome as estimated by densitometry (**Figure 1**). With the objective of identifying most of the components of this exoproteome, we analyzed the sample with the previously developed shotgun procedure [10]. The whole exoproteome was excised from a short SDS-PAGE migration (3 mm) as a single small polyacrylamide band. After trypsinization, the peptide mixture was injected into a C18 reversed phase chromatography column coupled to a high resolution LTQ-Orbitrap XL hybrid mass spectrometer. The acquired tandem mass spectra were assigned with the Mascot software using as database the complete list of annotated CDS from *Phaeobacter strain DSM 17395*. The dataset comprised 2321 MS/MS spectra, from which 1112 could be assigned to specific peptides from *Phaeobacter strain DSM 17395* (**Table S1 in File S1**). These spectra corresponded to 478 different peptides which validated the presence of 75 proteins with at least two different peptides (**Table S2 in File S1**). In terms of spectral counts, the first 15 proteins accounted for half of the total number of assigned spectra. The most detected proteins were an outer membrane porin (ZP_02147263), a flagellin-like protein (COG1344, ZP_02145485), a hemolysin-type calcium-binding protein (ZP_02144235), and the periplasmic phosphate-binding subunit of a phosphate ABC transporter (ZP_02143869) with 86, 55, 52, and 41 MS/MS spectra, respectively (**Table S2 in File S1**). Despite all these proteins were expected to be secreted, none was abundant enough (8% of total MS/MS spectra) as to be considered the highly secreted protein seen in **Figure 1**. Furthermore, their molecular weights (32, 28, 116, and 37 kDa, respectively) differ from that expected for this protein (≈55 kDa). Therefore, the shotgun results were in disagreement with the SDS-PAGE information and the abundant 55 kDa protein could not be identified. To solve this issue, we fully resolved the exoproteome onto a SDS-PAGE gel as shown in **Figure 1** and analyzed by nanoLC-MS/MS the protein content of the 55 kDa polyacrylamide band. Although only one major polypeptide was expected to be present in this polyacrylamide band, we evidenced 48 proteins identified with at least 2 peptides. **Table 1** reports the ten first proteins as ranked by their Mascot score (assigned MS/MS spectra and corresponding peptide characteristics are reported in **Table S3 in File S1**). Surprisingly we observed the protein GroEL Chaperonin (ZP_02145205) as the most detected protein with 61 MS/MS spectra although this protein is not expected to be secreted by bacteria. Another unexpected result was the presence in this polyacrylamide band of the 116 kDa protein ZP_02144235. Its tryptic sequence coverage showed that both the N- and C-termini were confirmed with proteomic-detected peptides, but the internal sequence was poorly covered (**Table S3 in File S1**). We presumed that this protein may be subjected to specific maturation such as a proteolytic cleavage. Again, none of the proteins shown in **Table 1** were in sufficient abundance as to be considered the abundant protein seen in **Figure 1**. Whether the expected abundant 55 kDa protein was encoded on the genome but missed during the annotation stage was also investigated by a new Mascot search using a six-frame translation of the whole nucleic acid sequence, as previously described [34]. This proteogenomic approach remained unsuccessful with no considerable findings (**Data not shown**). Therefore, the abundantly secreted protein of *Phaeobacter strain DSM 17395* remained unidentified with standard proteomic identification.

**Table 1:** List of the first ten proteins identified from the 55*P. gallaeciensis* DSM^1^.

Identification rank	Accession	Functional annotation	Number ofresidues	Protein Mascot Score	Molecularweight (Da)	Sequence Coverage (%)	Number of unique peptide	Spectral Count
1	ZP_02145205	Chaperonin GroEL	550	1632	57749	62	28	61
2	ZP_02146523	Oligopeptide/dipeptide ABC transporter*	518	929	57342	32	15	37
3	ZP_02144235	Hemolysin-type calcium-binding protein	1112	915	115931	15	13	35
4	ZP_02145689	Extracellular solute-binding protein, family 1	577	884	64572	36	15	33
5	ZP_02143510	Ser/Thr protein phosphatase/nucleotidase, putative	525	594	56013	36	12	25
6	ZP_02146184	Extracellular solute-binding protein, family 5	566	559	61051	31	11	21
7	ZP_02145456	Flagellar hook-associated protein	482	553	50979	28	9	16
8	ZP_02147263	Outer membrane porin	313	508	31831	28	9	21
9	ZP_02144479	Extracellular solute-binding protein, family 5	528	413	57617	17	7	20
10	ZP_02144175	Aconitate hydratase	895	431	96861	13	10	19

1 Detected with at least three different peptides.

*Periplasmic component.

### *De novo* Sequencing with High Resolution Data Revealed the Identity of the Abundantly Secreted Protein

**Figure 2:**
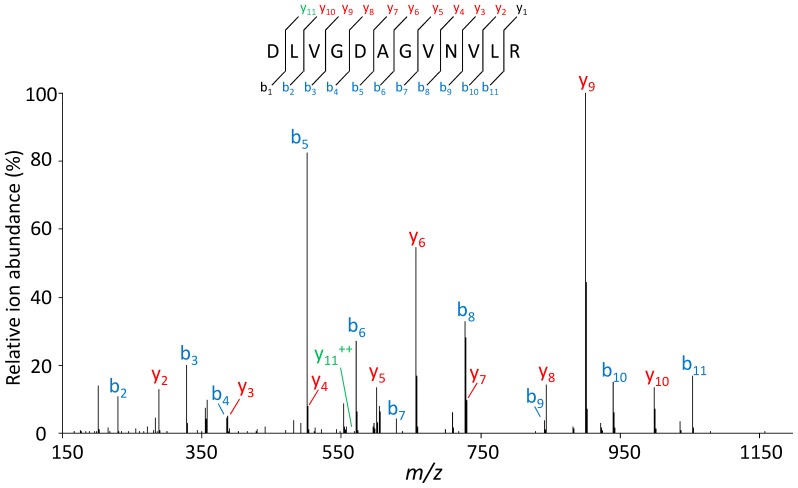
MS/MS spectrum of the semi-tryptic peptide [288–299] belonging to ZP_02147451. The MS/MS spectrum was acquired with a FT/FT procedure with an LTQ-Orbitrap XL hybrid mass spectrometer. The peptide sequence is shown on the top with the collision-induced fragmentation pattern. The *b* and *y* ions are shown in blue and red, respectively. The *y_11_* di-charged ion is labeled in green.

We carried out a search to manually assign spectra that had not been automatically assigned to a tryptic peptide comprised in the *Phaeobacter strain DSM 17395* CDS database. For this *de novo* sequencing strategy, we reanalyzed the peptides from the 55 kDa protein band by nanoLC-MS/MS with a FT/FT procedure which enables getting higher resolution MS/MS spectra and better mass accuracy (15,000 in the orbitrap analyzer instead of the 3,000 previously done with the LTQ analyzer). From this new dataset acquisition, 246 MS/MS spectra were assigned directly with the Mascot software using the complete list of annotated CDSs from *Phaeobacter strain DSM 17395* as a database. The same dataset was also searched with the *de novo* sequencing software Peaks Studio 5.3 identifying 258 new MS/MS spectra that had not been assigned by Mascot. We manually annotated these spectra checking all peptide sequence possibilities. **Figure 2** shows the annotation of a representative MS/MS spectrum with a *m/z* ratio measured at 614.33826 amu corresponding to a di-charged peptide with a semi-tryptic sequence, DLVGDAGVNVLR. Peak assignment was confident with the detection of 10 monoprotonated *b* ions and 9 monoprotonated *y* ions, except that the second and eleventh residues could be either leucine or isoleucine. We could also assign the first residue with the detection of the *y_11_* diprotonated fragment ion. A total of five sequences could be confidently assigned because of MS/MS mass accuracy. The Blast analysis of each of these five tentative peptides revealed strict identities to the ZP_02147451 protein from *Phaeobacter strain DSM 17395*. Curiously, all five *de novo* sequenced peptides corresponded to semi-tryptic peptides. With these data in hands, we finally supposed that the abundant exoprotein was ZP_02147451, annotated as a hemolysin-type calcium binding protein (RTX-toxin). Its theoretical molecular weight, 52624 Da, matched the expected molecular weight estimated from the SDS-PAGE gel.

### High Resolution Data Allowed a High Coverage of ZP_02147451 with Non-tryptic Peptides

**Figure 3:**
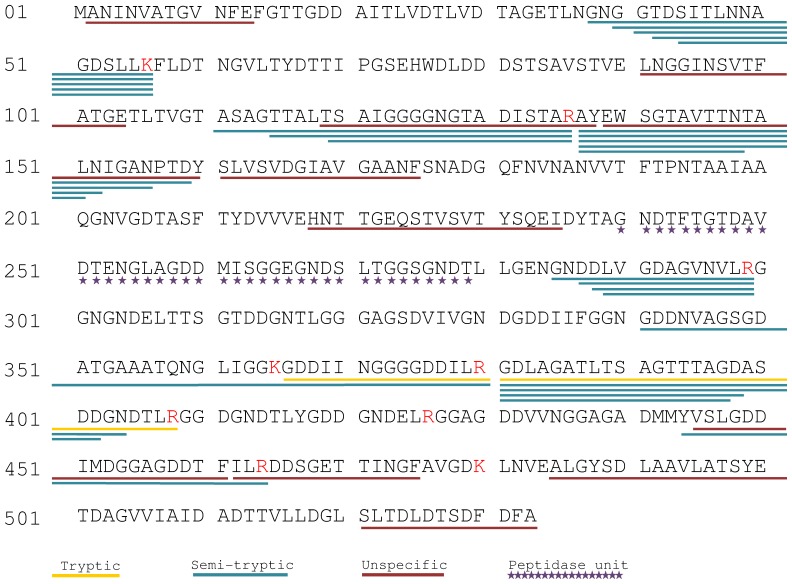
ZP_02147451 sequence coverage with non-tryptic, semi-tryptic, and tryptic peptides. The ZP_02147451 sequence is represented with its peptidase motif (residues 240 to 279) pointed out with purple stars. Peptides identified with the “no-enzyme” Mascot search are symbolized with a line underlining the sequence. Tryptic, semi-tryptic, and non-tryptic peptides are indicated in yellow, blue and red, respectively.

To confirm the identity of the protein and because *de novo* sequencing of some MS/MS spectra had shown the presence of abundant non-tryptic peptides, we re-analyzed the FT/FT experimental data with the Mascot software using “no-enzyme” as the enzyme parameter. **Table S4** **in File S1** lists the resulting 228 MS/MS spectra assigned to 128 distinct peptides belonging to proteins of the *Phaeobacter strain DSM 17395* bacterium. As expected, less MS/MS spectra are assigned with the no-enzyme search mode than with the trypsin search mode, due to the loss of discrimination introduced by the larger search space. This time the hemolysin-type calcium binding protein ZP_02147451 was clearly the most abundant protein identified accounting 67 MS/MS spectra assigned (30% of the spectral counts). A total of 36 distinct peptides were found for this protein and allowed 51% coverage of the whole sequence (**Figure 3**). According to this “no-enzyme” Mascot search, 24 peptides were semi-tryptic, 10 items do not arise from a specific cleavage, and only 1 is a tryptic peptide despite the trypsinization protocol applied. Peptides were evenly distributed along the protein sequence except the peptidase unit region spanning residues 240 to 279 (**Figure 3**). **Table 2** shows the comparative list of proteins assigned in the two Mascot search modes (“trypsin” or “no-enzyme”). In order to compare results obtained in the two search modes we defined the Enzyme Unspecificity Factor (EUF) for each protein as the number of unique peptides in the no-enzyme MASCOT search divided by the number of unique peptides in the trypsin MASCOT search. As seen in the table, ZP_02147451 is the only protein having a higher number of unique peptides (18-fold more) when the “no-enzyme” condition is used. MASCOT search with the “no-enzyme” parameter does not change significantly the results of spectral counting when cellular proteome are analyzed as based on our previous data (Data not shown) from another *Roseobacter*, namely *Roseobacter denitrificans* [35].

**Table 2:** List of proteins identified in the 55/FT proteomic procedure^1^.

			"Trypsin" as MASCOT search criterion					"No-enzyme" as MASCOT search criterion			
Accession	Functional annotation	Molecularweight (Da)	Protein Mascot Score	Sequence Coverage (%)	Number of unique peptides	Spectral Count	EUF$	Protein Mascot Score	Sequence Coverage (%)	Number of unique peptides	Spectral Count
ZP_02145205	chaperonin GroEL	57749	1118	50	22	38	**68**	905	37	15	20
ZP_02144235	Hemolysin-type calcium-binding protein	115931	685	13	12	48	**92**	711	15	11	40
ZP_02146523	oligopeptide/dipeptide ABC transporter*	57342	533	27	10	24	**70**	440	22	7	12
ZP_02145689	extracellular solute-binding protein, family 1	64572	354	19	7	12	**71**	293	13	5	7
ZP_02146184	extracellular solute-binding protein, family 5	61051	339	17	6	12	**83**	308	14	5	11
ZP_02144479	extracellular solute-binding protein, family 5	57617	338	18	6	10	**83**	302	15	5	6
ZP_02143510	Ser/Thr protein phosphatase/nucleotidase	56013	263	16	6	10	**83**	235	14	5	9
ZP_02143869	phosphate ABC transporter*	37145	221	9	2	3	**100**	221	9	2	3
ZP_02144175	aconitate hydratase	96861	232	9	6	9	**33**	112	3	2	2
ZP_02147263	outer membrane porin	31831	213	20	5	10	**40**	115	7	2	5
ZP_02145456	Flagellar hook-associated protein	50979	196	15	4	4	**75**	163	10	3	3
ZP_02145455	S-adenosyl-L-homocysteine hydrolase	45860	195	13	4	4	**100**	256	17	4	4
ZP_02143988	Hemolysin-type calcium-binding protein	30261	190	24	3	6	**100**	188	24	3	5
ZP_02144038	hypothetical protein RGBS107_15846	51273	152	12	4	8	**75**	127	7	3	5
ZP_02147451	Hemolysin-type calcium-binding protein	52624	163	8	2	4	**1800**	2463	51	36	67
ZP_02146962	extracellular solute-binding protein, family 1	47415	154	12	3	4	**67**	125	8	2	2

1 Detected with at least three different peptides. The values are from a representative experiment.

*Periplasmic component.

$ EUF stands for Enzyme Unspecificity Factor, defined as the ratio number of unique peptides (no-enzyme MASCOT search) per number of unique peptides (trypsin MASCOT search).

### The Recalcitrance of ZP_02147451 is Caused by an Extreme Lack of Basic Residues in its Sequence

The ZP_02147451 protein is a RTX-toxin with 533 amino acids that contains only a few basic residues (three lysines and six arginines). These residues are trypsin targets for proteolysis and, therefore, this RTX-like protein will derive in extremely large peptides after digestion. Considering the length of the protein and the position of these basic residues, only three tryptic peptides could be considered as detectable by the LC-MS/MS procedure with the LTQ-Orbitrap XL hybrid mass spectrometer (*m/z* range from 300 to 1800 th). When reconsidering all the peptides detected of ZP_02147451, less than half of the cleavages occurred at the C-terminus of arginines (30%) and lysines (12%) in accordance to the trypsin activity. Nevertheless, a large number of cleavages (20%) also arose at the C-terminus of asparagine (N). Other residues like glutamic acid (8%) and aspartic acid (7%) were also frequently found at the C-terminus of the detected peptides **(Figure S1)**. Thus, acidic residues frequently appeared at this position although trypsin could not explain such cleavages. Taken together, the low number of detectable tryptic peptides of the RTX-toxin ZP_02147451 is the reason why we observed other proteolytic derived peptides. These alternative proteolytic activities are usually rare and masked by the highly abundant tryptic peptides. Furthermore, in standard analysis these peptides would not be considered during the search.

We subjected the whole exoproteome of *Phaeobacter strain DSM 17395* to a novel shotgun analysis with the FT/FT procedure in order to apply a *de novo* sequencing approach and evaluate alternative proteolysis patterns. The data were analyzed with MASCOT using a “no-enzyme” or “trypsin” parameter. **Table 3** shows the comparative list of proteins assigned with at least five different peptides in the “no-enzyme” Mascot analysis mode. Interestingly, with “trypsin” as criterion for the Mascot search, ZP_02147451 was detected with only one unique peptide (2 spectral counts). The same protein was detected with 12 peptides when the “no-enzyme” parameter was used. ZP_02147451 was the only protein that exhibited such a large increase of detected peptides when the non-tryptic search mode was used (12-fold, **Table 3**). Noteworthy, we did not find any specific proteolytic pattern in the other proteins because of the small number of extra non-tryptic peptides detected during the analysis.

**Table 3:** List of proteins identified in whole exoproteome of *Phaeobacter strain DSM 17395* using the FT/FT procedure^1^.

			"Trypsin" as MASCOT search criteria						"No-enzyme" as MASCOT search criteria				
Accession	Functional annotation	Molecular weight (Da)	Protein Mascot Score	Sequence Coverage(%)	Number of unique peptides	Spectral Count	NSAF (%)	EUF$(%)	Protein Mascot Score	Sequence Coverage(%)	Number of unique peptides	Spectral Count	NSAF (%)
ZP_02144235	Hemolysin-type calcium-binding protein	115932	1109	20	17	70	2.4	88	1043	18	15	59	2.8
ZP_02145485	flagellin-like protein	28065	1057	66	16	63	9.0	106	1157	63	17	47	9.4
ZP_02146962	extracellular solute-binding protein, family 1	47415	888	46	13	34	2.9	92	853	44	12	32	3.8
ZP_02143869	phosphate ABC transporter*	37146	862	49	15	33	3.6	87	816	50	13	23	3.5
**ZP_02147451**	**Hemolysin-type calcium-binding protein**	**52624**	**82**	**3**	**1**	**2**	**0.2**	**1200**	**825**	**21**	**12**	**17**	**1.8**
ZP_02145205	chaperonin GroEL	57750	837	45	15	23	1.6	87	839	41	13	19	1.8
ZP_02147263	outer membrane porin	31831	798	34	12	156	19.7	108	848	36	13	100	17.6
ZP_02144302	glutathione synthetase	39420	622	46	10	20	2.0	80	544	34	8	17	2.4
ZP_02146401	phosphonate ABC transporter*	32128	539	49	10	19	2.4	80	513	35	8	14	2.4
ZP_02147202	TRAP dicarboxylate transporter	36347	548	38	9	23	2.5	89	522	33	8	22	3.4
ZP_02143908	TRAP transporter solute receptor	34084	541	35	11	26	3.1	82	498	35	9	16	2.6
ZP_02146891	Amino acid ABC transporter*	35345	512	41	9	16	1.8	89	471	41	8	14	2.2
ZP_02146522	Hemolysin-type calcium-binding region	31794	509	33	7	21	2.7	100	501	33	7	14	2.5
ZP_02146523	oligopeptide/dipeptide ABC transporter*	57342	499	26	9	18	1.3	78	441	21	7	14	1.4
ZP_02144316	putative iron ABC transporter*	35198	486	42	9	16	1.8	78	428	42	7	14	2.2
ZP_02146543	hypothetical protein RGBS107_00865	47171	479	31	8	17	1.5	75	430	23	6	12	1.4
ZP_02144531	OmpA/MotB	22083	434	82	9	11	2.0	56	325	48	5	6	1.5
ZP_02143879	Extracellular ligand-binding receptor	39919	408	47	8	14	1.4	75	337	36	6	9	1.3
ZP_02146376	putative sugar ABC transporter*	34311	399	41	8	19	2.2	75	345	41	6	12	2.0
ZP_02146184	extracellular solute-binding protein, family 5	61051	377	20	6	10	0.7	100	373	20	6	9	0.8
ZP_02144107	hypothetical protein RGBS107_16191	40145	368	38	7	11	1.1	86	341	28	6	10	1.4
ZP_02146027	EF hand domain protein	17617	363	53	6	10	2.3	67	294	36	4	7	2.2
ZP_02146810	zinc/manganese/iron ABC transporter*	34197	333	15	5	9	1.1	100	328	15	5	7	1.1
ZP_02145961	extracellular solute-binding protein, family 1	48328	322	15	4	6	0.5	125	366	20	5	7	0.8

1 Detected with at least three different peptides. The values are from a representative experiment.

*Periplasmic component.

$ EUF stands for Enzyme Unspecificity Factor, defined as the ratio number of unique peptides (no-enzyme MASCOT search) per number of unique peptides (trypsin MASCOT search).

### RTX-toxins Exhibit Unusually Low Content in Basic Residues

**Figure 4:**
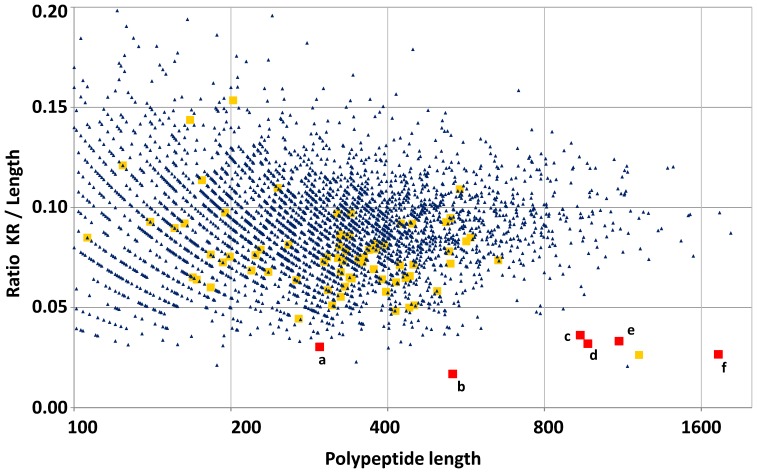
Occurrence of basic residues in proteins from *Phaeobacter strain DSM 17395.* The graph reports the ratio Lysine (K) and Arginine (R) residues per protein length of all the proteins with length above 100 residues encoded by *Phaeobacter strain DSM 17395*. Proteins are symbolized by a blue triangle. RTX-like proteins and the other exoproteins detected by tandem mass spectrometry are represented by red and yellow squares, respectively. The ZP_02143988.1 (a), ZP_02147451.1 (b), ZP_02144693.1 (c), ZP_02146960.1 (d), ZP_02144235.1 (e), and ZP_02146514.1 (f) RTX-toxins are indicated. Only the ZP_02143988.1 RTX-like protein has been detected by mass spectrometry.

After identifying the RTX-like protein ZP_02147451 by *de novo* sequencing we further investigated the reason of its recalcitrance to our standard proteomic approach. Within the 533 residue protein we only found nine basic amino acids. Thus, it results refractory to standard trypsin-based proteomic approaches because of the large tryptic peptides generated. These cannot be analyzed properly by mass spectrometers operated in classical conditions. We checked whether this was a specific feature for this protein compared to the whole *Phaeobacter strain DSM 17395* theoretical proteome. **Figure 4** shows the comparison for all the proteins with length above 100 residues. Indeed, ZP_02147451 is by far unusual in terms of basic residue content, *i.e.* 1 basic residue every 59 residues while the mean value is every 11 residues within the whole theoretical proteome of *Phaeobacter strain DSM 17395*. Noteworthy, we detected that other RTX-toxins encoded on the *Phaeobacter strain DSM 17395* genome are also unusually poor in basic residues. ZP_02146514, ZP_02146960, ZP_02146988, ZP_02146235, and ZP_02146693 are large RTX-toxin proteins (1725, 969, 296, 1112, and 937 amino acids respectively) which contain 1 basic residue every 30 residues in average (**Figure 4**). We wondered whether this characteristic is conserved among the other secreted proteins. **Figure 4** shows that the other proteins detected in the exoproteome of *Phaeobacter strain DSM 17395* do not exhibit such a specific trend, although most have a slightly lower RK content compared to the average. This trait is a common issue within the RTX-like proteins coded in *Roseobacter* members. The analysis of the 107 RTX-like proteins coded in 12 other sequenced *Roseobacter* strains revealed one basic residue every 27 amino acids (Data not shown). As an example, *Roseobacter* sp. MED193 exhibits two RTX-like proteins, ZP_01058551 and ZP_01057949, with an average tryptic peptide size of 80 and 73 residues, respectively. As a result, the secreted RTX-like proteins are generally refractory to trypsin digestion. We evidenced as illustrated in Figure 4 the presence in the exoproteome of a large protein with a low ratio KR/polypeptide length through the identification of three distinct peptides. This protein, ZP_02146938.1, is large (122 kDa) and annotated as the hypothetical protein RGBS107_08210. Its low content in basic residues and large molecular weight may suggest that it could also be a “RTX-toxin” far-related protein. Indeed, a Blast analysis confirmed such relationship.

### Concluding Remarks

Secreted proteins play an essential role in successful adaptation of bacteria to their environment. These exoproteins are crucial for nutrient import, motility, and biofilm formation. In some cases, they are used by pathogens to adhere to and degrade cell walls from their targets and to suppress defense responses from their hosts [36]. With the development of novel high-throughput proteomic strategies and the use of novel generation of hybrid tandem mass spectrometers, novel insights were recently obtained regarding the secretome from various marine *Roseobacter* strains in several physiological conditions [10], [12]. Here, we reported the analysis of the exoproteome of another *Roseobacter* member, namely *Phaeobacter strain DSM 17395*, which is known to produce potent and selective algaecides [25]. In this case, we found that the major secreted protein, representing 60% of the total exoproteome, was refractory to classical proteomic approach identification. This challenging issue made us changes our strategy and resort to *de novo* sequencing in order to identify the polypeptide. Interestingly, we found that the abundantly secreted protein was a RTX-like toxin, namely ZP_02147451, annotated as a hemolysin-type calcium-binding protein. This 533 amino acids polypeptide comprised a domain spanning from amino acids 239 to 279 and shared high similarities to the C-terminal peptidase M10 serralysin. Noteworthy, we did not find any specific proteolytic pattern for the other proteins because of the small number of extra non-tryptic peptides detected during the analysis. This indicates that no massive proteolysis occurred during exoproteome extraction as previously experienced [7], [12], [37]. As previously reported, the presence of highly secreted RTX-toxins seems to be a common issue in some *Roseobacter* strains [12]. In this sense, the main component of the exoproteome from *Ruegeria pomeroyi* DSS-3 was the PaxA RTX-toxin, observed whatever the culture condition experimentally tested. While some RTX-like proteins have been well characterized and considered as the main virulence factors in several well-known uropathogenic strains or different pathogenic *Vibrio* species [38] most of these toxin-like proteins remain uncharacterized in nonpathogenic environmental strains. This diverse group of RTX-like secreted proteins encoded in *Roseobacter* members is thought to have a potential effect on other members of the marine community in order to capture organic matter [39]. Interestingly, the secreted RTX-like proteins were depleted from the exoproteome of *R. pomeroyi* DSS-3 only in presence of a natural marine port community indicating a potential effect on its environment [7].

We concluded that the low content in basic residues detected in RTX-like proteins from *Roseobacter* strains could be linked to the function of these proteins rather than to the fact that they are secreted. Because these proteins encompass peptidase domains, their content in basic residues could be constrained to a low level for a higher resistance to the proteolysis of trypsin-like proteases or autoproteolysis as RTX-toxins may autoprocess [40], [41]. Because they are secreted to the marine environment in high amounts, at least ZP_02147451 from *Phaeobacter strain DSM 17395* and PaxA from *R. pomeroyi* DSS-3, their function should be vital for bacteria from the *Roseobacter* clade. The present study shows that usual proteomic parameters for the interpretation of MS/MS data are misleading when exoproteomes are being analyzed. Here, trypsin-specific searches for MS/MS assignment did not identify correctly the ZP_02147451 RTX-toxin and led to false quantitative results regarding the main exoproteome components. Therefore, we propose for further exoproteome studies to analyze by tandem mass spectrometry the proteins cleaved with a combination of different proteases (*e.g.* trypsin, chymotrypsin or EndoGluC proteases) in order to not underestimate, or simply miss, abundantly secreted proteins of interest. As an example, here the experimental proteolysis of the 55 kDa band with chymotrypsin allowed the identification of the ZP_02147451 RTX-toxin with 701 MS/MS spectra assigned to the protein out of 817 MS/MS spectra recorded, i.e. 85% % (**Table S5 in File S1**). Proteolysis with endo glu-C led to the identification of the same protein with 662 assigned MS/MS spectra out of 747 MS/MS spectra recorded, i.e. 89 (**Table S6 in File S1**). In this paper we outlined a strategy that could be systematically used to identify secreted proteins in environmental environments. It would be worth re-analyzing previous exoproteome datasets to check whether the lack of identification of an intense protein was common to several of these studies. However, the lack of the corresponding raw data (SDS-PAGE and MS/MS datasets) prevented such analysis. While RTX-proteins are found numerous in marine bacteria with more than a thousand members, some have been found involved in virulence of uropathogenic *E. coli* revealing strong medical interest [42]. Our results point at the specific structural characteristics of these proteins as basic residues have been counter-selected during evolution.

## Supporting Information

Figure S1 **Relative abundance of C-ter cleaved residues within the whole mass spectrometry-detected ZP_02147451 peptides.**
(TIF)

File S1 **Table S1, List of tryptic peptides identified in the whole exoproteome from** ***Phaeobacter strain DSM 17395*** **using a standard shotgun procedure. Table S2, List of proteins identified in the whole exoproteome from** ***Phaeobacter strain DSM 17395*** **using a standard shotgun procedure. Table S3, List of tryptic peptides identified in the 55 kDa SDS-PAGE band of the exoproteome from** ***Phaeobacter strain DSM 17395*** **using a standard shotgun procedure. Table S4, List of peptides identified in the 55 kDa band of the exoproteome from** ***Phaeobacter strain DSM 17395*** **using the FT/FT procedure and with "no-enzyme" as MASCOT search criteria. Table S5, List of peptides identified in the 55-kDa band of the exoproteome from** ***Phaeobacter strain DSM 17395*** **after chymotrypsin digestion using the FT/FT procedure. Table S6, List of peptides identified in the 55-kDa band of the exoproteome from** ***Phaeobacter strain DSM 17395*** **after endo-GluC digestion using the FT/FT procedure.**
(XLSX)
