# Supplementary figures and images for: Assessing the Exoproteome of Marine Bacteria, Lesson from a RTX-Toxin Abundantly Secreted by Phaeobacter Strain DSM 17395
Source: PLoS One. 2014 Feb 24;9(2):e89691. doi: 10.1371/journal.pone.0089691 (PMC3933643; doi:10.1371/journal.pone.0089691)

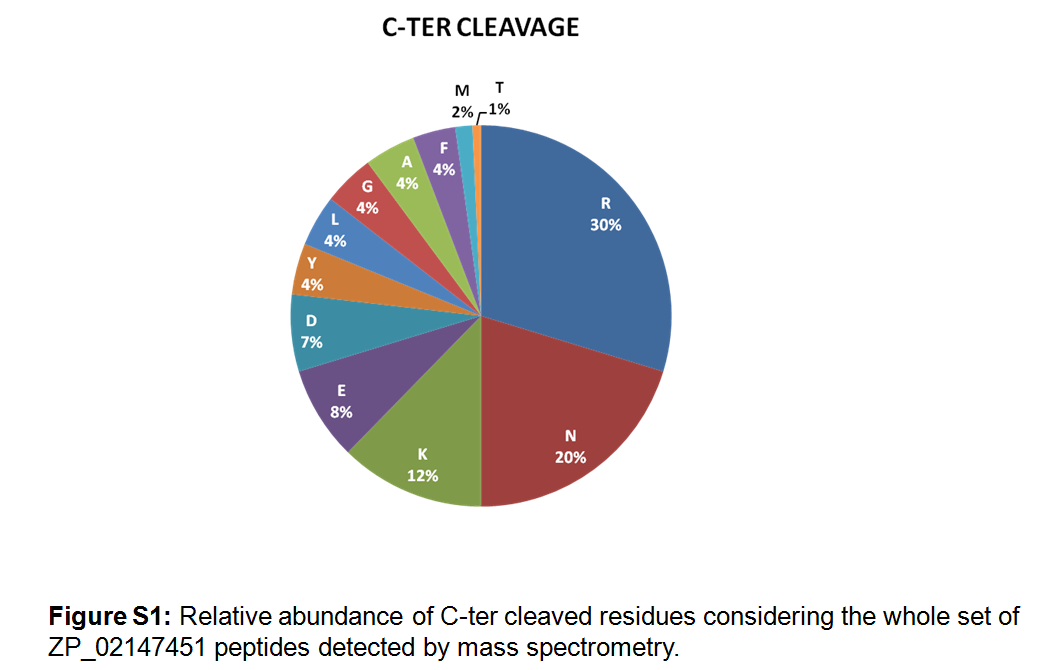

Supplement: Figure S1 — Relative abundance of C-ter cleaved residues within the whole mass spectrometry-detected ZP_02147451 peptides. (TIF) [file pone.0089691.s001.tif]
